# Supplementary figures and images for: Dharma: A novel, clinically grounded machine learning framework for pediatric appendicitis—Diagnosis, severity assessment and evidence-based clinical decision support
Source: PLOS Digit Health. 2026 Jan 21;5(1):e0000908. doi: 10.1371/journal.pdig.0000908 (PMC12822984; doi:10.1371/journal.pdig.0000908)

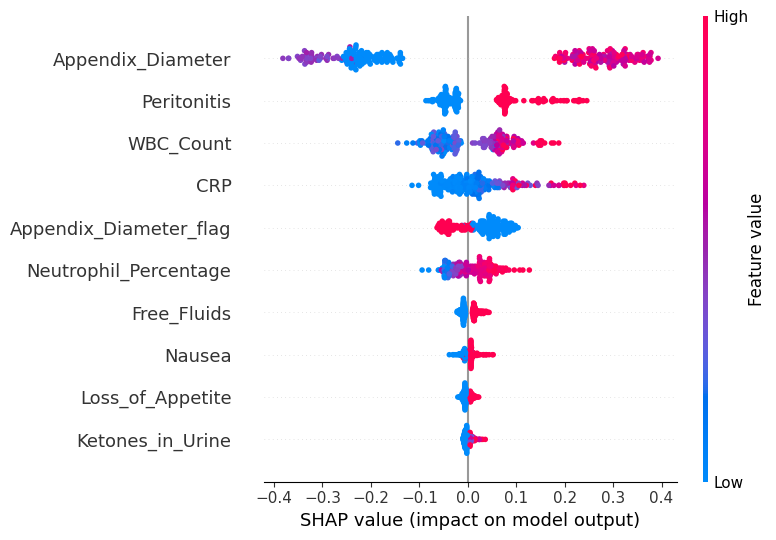

Supplement: S1 Fig — (TIF) [file pdig.0000908.s001.tif]

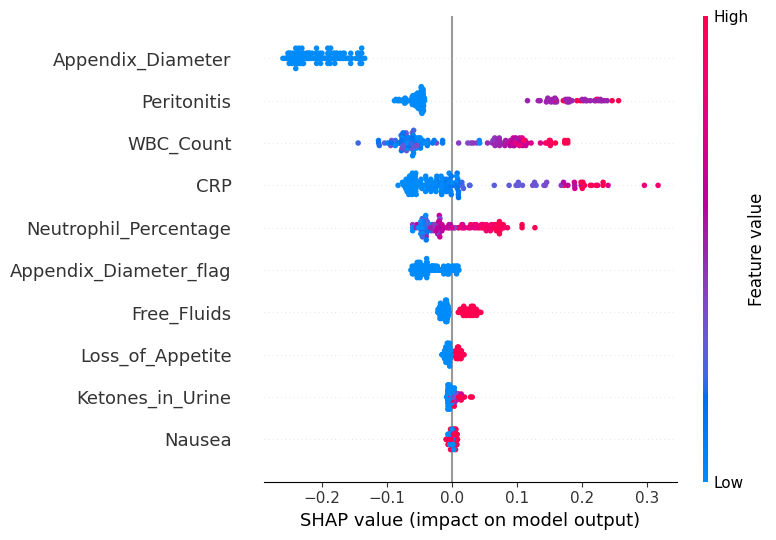

Supplement: S2 Fig — (TIF) [file pdig.0000908.s002.tif]

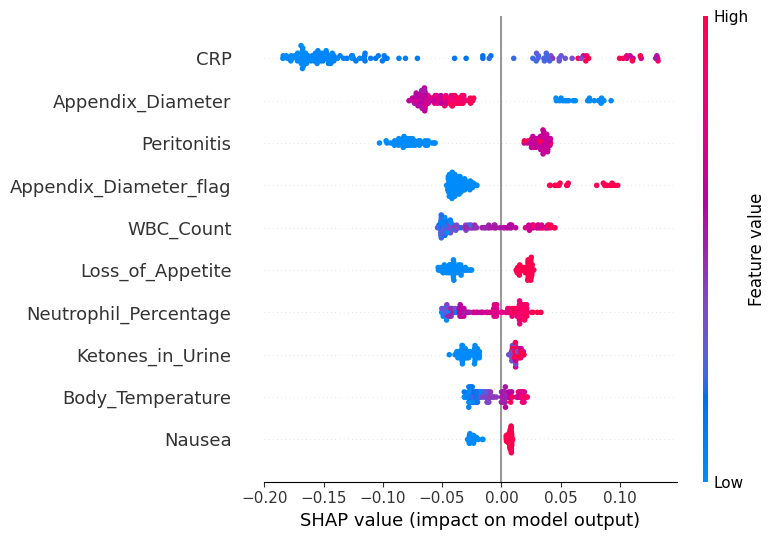

Supplement: S3 Fig — (TIF) [file pdig.0000908.s003.tif]

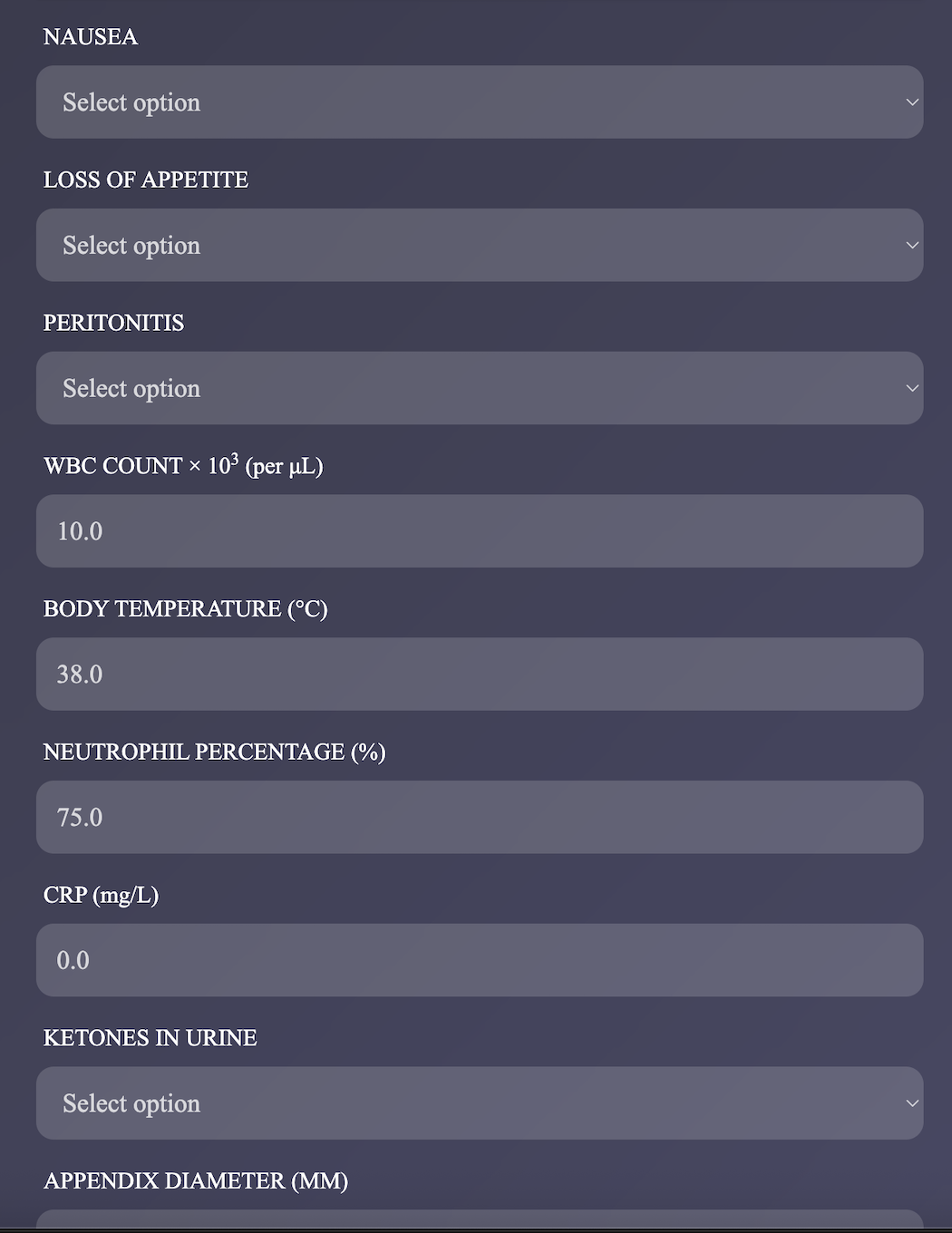

Supplement: S5 Fig — (TIF) [file pdig.0000908.s005.tif]

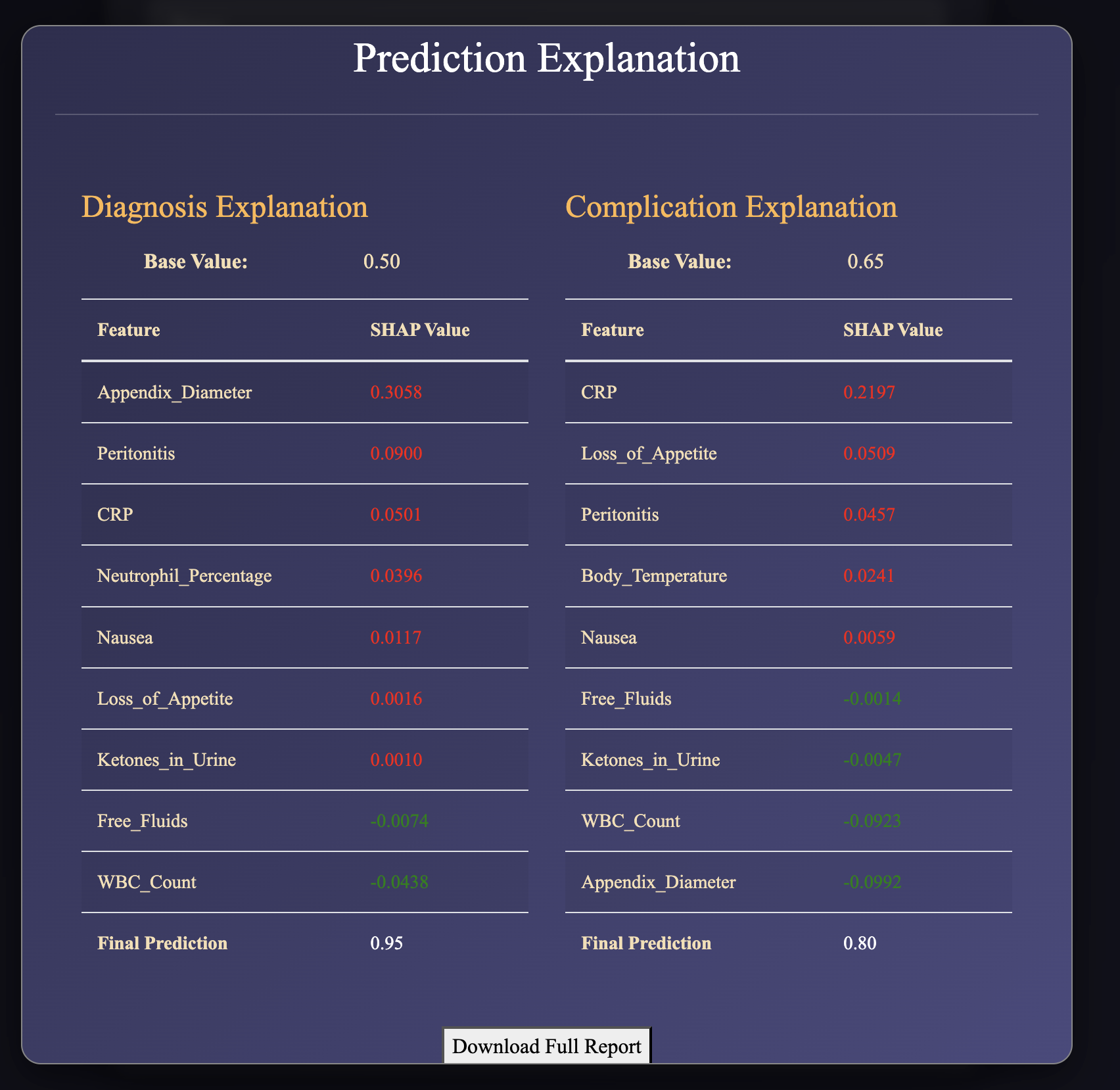

Supplement: S7 Fig — (TIF) [file pdig.0000908.s007.tif]
