# Supplementary material for: Dharma: A novel, clinically grounded machine learning framework for pediatric appendicitis—Diagnosis, severity assessment and evidence-based clinical decision support
Source: PLOS Digit Health. 2026 Jan 21;5(1):e0000908. doi: 10.1371/journal.pdig.0000908 (PMC12822984; doi:10.1371/journal.pdig.0000908)
Supplement: Attachment — Submitted filename: Response to Reviewers.docx [file pdig.0000908.s011.docx]

Manuscript ID: PDIG-D-25-00316

Title: Dharma: A novel machine learning framework for pediatric appendicitis—diagnosis, severity assessment and evidence-based clinical decision support

Journal: PLOS Digital Health

Dear Dr. Ghasemi,

Thank you for your interest in our project and for taking the time to provide feedback. We greatly appreciate the opportunity to strengthen our work and make it more scientifically rigorous.

In our revision we have made the following major changes:

- We benchmarked Dharma against state-of-the-art tabular machine learning models, XGBoost and LightGBM.
- Instead of imputing missing variables with arbitrary zeros, we developed a custom, clinically grounded, intelligent imputer, **Dharma_Imputer**. Details are provided below in response to Reviewer 2, Comment 3.
- Reported 95% confidence intervals via bootstrap resampling

**Responses to Reviewers’ Comments:**

Thank you to the reviewers for investing their time in evaluating our manuscript and providing constructive feedback. Below, we address each comment point by point, detailing the revisions made in response and highlighting how these changes strengthen the manuscript.

**Reviewer 1**:

**Comment 1**: Inadequate data source and representativeness: The main data were from the Children's Hospital of Regensburg, Germany. Although literature data were supplemented, the lack of validation in multi-center and different regions may affect the universality of the model in resource-constrained areas.

**Response**: We acknowledge that the primary dataset was derived from a single center, which limits direct evidence for generalizability. This limitation has been addressed in lines 612–614 of the revised manuscript. Nonetheless, the clinical features and biomarkers used in Dharma—including CRP, WBC count, neutrophil percentage, and urinary ketones—are globally standardized in the evaluation of pediatric appendicitis, which supports their applicability across diverse populations.

Although multi-center primary data collection was not feasible at this stage, we rigorously validated Dharma through 555 bootstrap resamples with 10-fold cross-validation, achieving an AUC-ROC of 0.98 [0.97–0.99], which provides preliminary support for generalizability. Furthermore, Dharma was specifically designed for use in resource-constrained settings. For example, among its 10 core features, variables that are difficult to obtain in such environments (e.g., CRP, urinary ketones, free fluid) are handled using our clinically informed **Dharma_Imputer**. Likewise, radiological features such as appendix diameter—which may often be unavailable—are addressed with a missingness indicator column, enabling the model to treat non-visualization as a predictive signal. Notably, this design preserved excellent performance, with an AUC-ROC of 0.96 [0.93–0.99] even in cases without appendix visualization.

**Comment 2**: Supplement systematic comparisons with existing ML tools to clarify technical advantages: Only the Random Forest model was used without systematic comparisons with other mainstream ML algorithms (such as GBM, XGBoost, CatBoost), making it difficult to verify the universality of the model's superiority.

**Response**: For the diagnostic task, we benchmarked Dharma against XGBoost and LightGBM. For the prognostic task, we compared Dharma with XGBoost under two settings: (i) simple hyperparameters and (ii) highly regularized hyperparameters optimized for recall. Each comparator model was tuned to its best performance via grid search in cross-validation. These details are now described in the Model Evaluation subsection of Methods, the Benchmarking Analyses subsection of Results, and lines 560–570 of the Discussion.

**Comment 3**: No images provided.

**Response**: In the revised manuscript, we have added SHAP dot plots illustrating the diagnostic and prognostic performance of Dharma (Supplementary Figures S1–S3). Additionally, we have included images of the developed web tool (Supplementary Figures S4–S6).

**Comment 4**: The titles of Table 3 and Table 4 are separated from the tables. Some literature citations lack academic DOI numbers (such as [8], [35], [44], [45], [46]), and some literature citations lack page ranges.

**Response**: We have cleaned and reformatted the tables as requested. Reference [44] directs to the publicly available site hosting the primary dataset of our study, while [46] links to our GitHub repository where the Dharma source code is published. For references [8], [35], and [46], the original articles were not retrievable; therefore, we have cited their corresponding PMIDs instead.

**Reviewer 2**:

**Comment 1**: The paper would benefit if you report your CV metric results along with 95% CI, which gives better readability than SD.

**Response**: We are thankful for your constructive comment. We have reported 95% confidence intervals using 555 bootstrap samples, with mean estimates for the key metrics (AUC-ROC, Sensitivity, Specificity, PPV, NPV, and Accuracy) calculated across 10-fold cross-validation within each bootstrap sample. This is presented in the *Dharma* subsection of the diagnostic results and in the prognostic results subsection. Additionally, we provide the full set of performance metrics for the SOTA models and conventional linear scoring tools, along with their comparisons based on 95% confidence intervals.

**Comment 2**: The tables need some tidying, for eg in Table 9. Why do you omit reporting for specificity, NPV, PPV in the 10-fold CV results? These should be added since only the CV results are what best predicts the real world model performance.

**Response**: We have cleaned and reformatted the tables as requested.

**Comment 3**: In the missing value imputation strategy it is stated that null values are imputed with zeros, is this only for categorical variables or all variables? Because, zero is an actual and clinically meaningful value for several variables (e.g., CRP = 0 mg/L). It would be better to replace it with an evidence-based imputation method (multiple imputation or model-specific missingness indicator). Report per-variable missingness and confirm performance stability.

**Response**: We are extremely grateful for your insightful feedback. Initially, we had imputed all variables using zero imputation; upon reflection, we recognized the limitations of this approach. To address this, we developed a custom imputer, the **Dharma_Imputer**. In this framework, inflammatory markers (WBC count, neutrophil percentage, CRP, and body temperature) were pooled and iteratively imputed using a decision tree regressor trained on all available data. Categorical variables were imputed with feature-specific decision tree classifiers. Missingness in the appendix diameter column was treated as an informative signal: a missingness indicator (missing_flag) was added, and null values were encoded as –1 placeholders.

All steps of this procedure were implemented within the **Dharma_Imputer**. The per-variable missingness is reported in *Supplementary Table 1*. We also evaluated alternative combinations of imputation strategies for continuous and categorical variables, including mean/mode imputation, KNN imputation, and linear regression–based iterative imputation. Across methods, no statistically or clinically significant differences in discriminatory performance were observed, confirming the stability of Dharma’s predictive ability irrespective of imputation approach (see lines 316–330, *Model Evaluation* section, Materials and Methods).

**Comment 4**: I think you should also address the limits and trade-offs of using SMOTE in the study's limitations section

**Response**: Thank you for this valuable comment. While drafting the limitations section regarding SMOTE, we recognized the risks of overfitting and signal dilution associated with synthetic oversampling. Consequently, we decided not to apply SMOTE in our work. Instead, for the complications model, we augmented the training data with 49 real, curated complicated appendicitis cases extracted from the literature. To address class imbalance, we applied NearMiss undersampling to reduce the negative class and further reinforced the bias by adjusting the class weights to a ratio of 1:5 in favor of the positive class. This strategy is detailed in lines 272–289 of the *Model Development* section in the Materials and Methods.

**Comment 5**: You only train a random forest model, the paper would benefit if you also train and compare it with other SOTA tabular data models, e.g. Xgboost or LightGBM + maybe also a deeplearning based model like TabNet.

**Response**: For the diagnostic task, we benchmarked Dharma against XGBoost and LightGBM. For the prognostic task, Dharma was compared with XGBoost under two conditions: (i) simple hyperparameters and (ii) highly regularized hyperparameters optimized for recall. Each comparator model was tuned to its optimal performance through grid search with cross-validation. These additions are now described in the *Model Evaluation* subsection of Methods, the *Benchmarking Analyses* subsection of Results, and lines 560–570 of the Discussion.

We did not include TabNet experiments, as our available training datasets for both diagnostic and prognostic models were far below the scale generally required for reliable training of deep learning–based tabular models. Applying TabNet under such constraints would likely yield unstable and uninformative results.

We are grateful to the reviewers and editors for their thoughtful and constructive feedback, which has helped us to substantially improve the clarity, rigor, and overall quality of our manuscript. We believe the revisions have strengthened the work and addressed all concerns. We remain at the reviewers’ and editors’ disposal for any additional clarifications.

Sincerely,
Anup Thapa, MBBS
Email: ajungthapa11@gmail.com
