# Supplementary material for: Dharma: A novel, clinically grounded machine learning framework for pediatric appendicitis—Diagnosis, severity assessment and evidence-based clinical decision support
Source: PLOS Digit Health. 2026 Jan 21;5(1):e0000908. doi: 10.1371/journal.pdig.0000908 (PMC12822984; doi:10.1371/journal.pdig.0000908)
Supplement: Attachment — Submitted filename: Response_to_Reviewers.docx [file pdig.0000908.s012.docx]

Manuscript ID: PDIG-D-25-00316

Title: Dharma: A novel machine learning framework for pediatric appendicitis—diagnosis, severity assessment and evidence-based clinical decision support.

Journal: PLOS Digital Health

Dear Dr. Ghasemi,

We are sincerely grateful for the continued support and guidance provided by the editorial team throughout the review process. Your input has been invaluable in helping us strengthen the scientific rigor, clarity, and overall quality of our manuscript. Below, we provide our detailed responses to the reviewers’ comments.

**Reviewer 2:**

We thank Dr. Josip Vrdoljak for the time and effort devoted to evaluating our work and for the constructive feedback that greatly improved our project. We are particularly appreciative of the comment on our imputation strategy, which ultimately led to the development of the clinically grounded *Dharma Imputer*, now also incorporated into the updated web application.

**Reviewer 3:**

**Comment 1:** Any inclusion/exclusion criteria mentioned for the inclusion of the sample in the Methods section.
**Comment 2:** Basis for sample size selection and sampling methods.

**Response:** We have now separated the *Data Description* and *Data Preprocessing* sections within *Materials and Methods*. The concerns regarding inclusion/exclusion criteria, sample selection, and sampling approach have been addressed in the *Data Description* subsection (lines 142–165) of the revised manuscript.

**Comment 3:** Other AI or machine learning models that have been researched for appendicitis—whether any comparison of your findings with them exists in the Discussion section.
**Comment 4:** Any superiority this model or the study findings have over the Alvarado score or other clinical scoring systems used for diagnosing appendicitis.

**Response:** Thank you for these valuable comments. We have expanded the Discussion to include comparisons with previously published machine learning models as well as traditional scoring systems. These revisions can be found in lines 560–579 of the updated manuscript.

We remain deeply grateful to the Editor, the PLOS Digital Health editorial team, and all reviewers for their insightful comments and guidance. Their feedback has greatly strengthened Dharma, helping us make it as scientifically rigorous and technically robust as it is clinically inspired.

Thank you once again.

Sincerely,

Dr. Anup Thapa, MBBS

Email: [ajungthapa11@gmail.com](mailto:ajungthapa11@gmail.com)

[anup.thapa@dharma-ai.org](mailto:anup.thapa@dharma-ai.org)
