# Supplementary material for: Dharma: A novel, clinically grounded machine learning framework for pediatric appendicitis—Diagnosis, severity assessment and evidence-based clinical decision support
Source: PLOS Digit Health. 2026 Jan 21;5(1):e0000908. doi: 10.1371/journal.pdig.0000908 (PMC12822984; doi:10.1371/journal.pdig.0000908)
Supplement: Attachment — Submitted filename: Response_to_Reviewers_auresp_3.docx [file pdig.0000908.s013.docx]

Manuscript ID: PDIG-D-25-00316

Title: Dharma: A novel, clinically grounded machine learning framework for pediatric appendicitis—diagnosis, severity assessment and evidence-based clinical decision support.

Journal: PLOS Digital Health

Dear Dr. Ghasemi,

We are extremely grateful for the continued support and guidance of the editorial team and reviewers, which has been invaluable in helping us clarify and more effectively communicate the aims and contributions of our work.

In line with the revisions, we have also updated the title and the author summary.

Below, we provide detailed responses to Reviewer 4’s comments.

**Comments 1, 3 and 7:**

We have now explicitly explained the rationale for using a 5:1 class weight and the higher SHAP base value (0.73) in the prognostic model, to prevent any misunderstanding of the term “bias” and to clearly communicate the reasoning behind these design choices. The manuscript has been revised as follows:

- **Model Development (Materials and Methods, Lines 274–283):** Details the deliberate use of asymmetric class weighting to minimize false-negative predictions for complicated appendicitis.
- **SHAP Analysis (Results, Lines 497–505):** Clarifies that the elevated base value reflects a conservative prognostic posture aligned with asymmetric clinical risk tolerance.
- **Discussion (Lines 529–555):** Interprets the cross-validation results in the context of medical reasoning, highlighting the clinical rationale behind these design choices.

**Comment 2, 4 and 5**:

We sincerely thank the reviewer for these insightful and constructive comments, which helped us better contextualize *Dharma* within the broader landscape of artificial intelligence and machine learning in medical diagnostics and prognostics.

In response, we have expanded both the Introduction and Discussion to more clearly situate our comparisons within the evolving paradigm of AI in medicine. Specifically, we now emphasize that while the referenced models represent powerful prediction-oriented architectures, *Dharma* was intentionally designed as a clinically grounded, hybrid framework—integrating domain knowledge, deliberate class weighting, and model interpretability—to support consequence-aware clinical reasoning rather than prediction alone. This clarification underscores that the task addressed by *Dharma* extends beyond conventional classification to the practical demands of real-world clinical decision-making.

We have also incorporated discussion informed by the suggested reference, *“COVID-19 Pandemic: A Worldwide Critical Review with the Machine Learning Model-Based Prediction.”* This work offers valuable insights into how mathematical modeling, domain expertise, and machine learning can be meaningfully integrated for disease prediction and outcome estimation. Building on these emerging concepts of domain-knowledge–guided machine learning, we now explicitly position **Dharma** within the continuum of hybrid AI approaches that bridge statistical learning with clinical reasoning.

Collectively, these revisions enhance conceptual clarity, strengthen the theoretical foundation of the manuscript, and more clearly articulate how *Dharma* contributes to contemporary AI-enabled clinical decision support.

We believe these revisions address the reviewers’ concerns and strengthen the manuscript.

Thank you once again for your continued support throughout this journey, which has been instrumental in helping us refine the clarity, rigor, and scientific coherence of our work.

Sincerely,

Dr. Anup Thapa Kshetri, MBBS

Email: [ajungthapa11@gmail.com](mailto:ajungthapa11@gmail.com)

[anup.thapa@dharma-ai.org](mailto:anup.thapa@dharma-ai.org)
